# Using the socioecological model to explore factors associated with obesity among reproductive age women

**DOI:** 10.3389/fpubh.2025.1498450

**Published:** 2025-02-05

**Authors:** Amanda Gilbert, Alicia Persaud, Sarah Farabi, Cindy Schwarz, Debra Haire-Joshu, Rachel G. Tabak

**Affiliations:** ^1^Prevention Research Center in St. Louis, Brown School at Washington University in St. Louis, St. Louis, MO, United States; ^2^Goldfarb School of Nursing at Barnes-Jewish College, St. Louis, MO, United States; ^3^Center for Human Nutrition, Washington University School of Medicine, St. Louis, MO, United States; ^4^Center for Diabetes Translation Research at Washington University in St. Louis, St. Louis, MO, United States

**Keywords:** Obesity, prevention, women, health behaviors, ecological

## Abstract

**Introduction:**

Women of reproductive age (18–44 years) are at an increased risk of developing obesity due to pregnancy, life-transitions, and marginalization. Obesity in women negatively affects women’s health and pregnancy outcomes and can increase risk their children will develop obesity. Less is known about obesity risk at the interpersonal and environmental levels for women of reproductive age. This study uses the socioecological model to explore women’s obesity risk across ecological levels.

**Materials and methods:**

A secondary cross-sectional analysis was conducted using baseline data (March 2019–June 2022) from the cluster-randomized Healthy Eating and Active Living Taught at Home (HEALTH) Dissemination and Implementation study. Descriptive statistics and multivariate logistic regression models were used to determine associations between individual, interpersonal, and environmental level factors with weight status (overweight vs. obesity).

**Results:**

Among 221 participants (43% Hispanic/Latino, 51% High school or less), 37% were overweight and 63% had obesity. Interpersonal and environmental factors were not statistically significantly associated with obesity relative to overweight in bivariate analyses. In multivariate models, individual level factors of high/moderate physical activity (OR = 0.47, 95% CI: 0.26,0.84, *p* = 0.01) and food insecurity (OR = 2.51, 95% CI: 1.33,4.71, *p* = 0.00) were statistically significantly related to risk of having obesity compared to being overweight.

**Discussion:**

Physical activity and food insecurity were associated with obesity in this study. Associations with interpersonal and environmental level factors were not statistically significant, which may be due to limited sample size or measures available to assess these levels. Future studies should investigate structural determinants (e.g., economic, neighborhood and physical environment), which may drive physical activity and food insecurity.

## 1 Introduction

Women of reproductive age (18–44) are at a greater risk for weight gain and obesity and experience disparities in obesity leading to health inequities (1–4). Women in this age group face unique obesity risks due to stresses such as pregnancy, parenthood, life transitions (e.g., leaving home, jobs, marriage), and social disadvantage (5–9). As of 2022, 33% of women aged 18–44 years old had obesity, with the age-adjusted prevalence of severe obesity (BMI > 30 kg/m^2) among adult women at 13%, double the rate for men (2, 10). Further, Black and Hispanic women age 20 and older experience higher rates of obesity at 57 and 44% respectively, compared to white women (40%) (1). These disparities are a result of systemic and historical racism that impact social determinants of health (SDOH) leading to higher social needs including chronic stress, unequal access to nutrient-dense foods and safe places to engage in physical activity, and fewer economic and social resources (3, 5, 11–16). High rates of obesity and disparities across race and ethnicity have health equity implications since obesity is associated with adverse health outcomes such as Type 2 diabetes mellitus, hypertension, dyslipidemia, coronary heart disease, chronic obstructive pulmonary disease, and poor pregnancy outcomes (17–20).

Healthy Eating and Active Living Taught at Home (HEALTH) is an effective, evidence-based intervention for obesity prevention among women of reproductive age (21). HEALTH embeds healthy eating and active living content adapted from the Diabetes Prevention Program into the Parents as Teachers (PAT) national home visiting organization, which has significant reach among women from pregnancy until the child is in kindergarten (22). Dissemination and implementation of HEALTH through The Healthy Eating and Active Living Taught at Home Dissemination and Implementation study (HEALTH D&I) holds promise for impacting the secular trends described above through the prevention of weight gain and promotion of weight loss (23). Examining data collected at baseline of the HEALTH D&I study provides a unique opportunity to explore factors associated with weight gain among women of reproductive age. In particular, the geographic, ethnic, and socioeconomic diversity of participants in the trial offers a unique opportunity to fill a gap in exploring these relationships in a group underrepresented in research.

To explore obesity risk factors within baseline data from HEALTH D&I, this analysis will utilize the socioecological model which represents the ecological theory of a particular health behavior or outcome (24). It demonstrates how several factors that interact at both macro- and micro-levels affect a person’s health and well-being (25). Socioecological theory is conceived in obesity research as being influenced by factors on the individual (e.g., genetics, health behaviors), interpersonal (e.g., parent–child relationship, family support), and neighborhood/community or environmental level (e.g., food availability, safe places to engage in physical activity) (26). The socioecological model aids in considering health equity when exploring obesity risk factors within this population. It underscores multiple levels of influence on obesity risk and the importance of looking at drivers of obesity risk beyond the individual level. Therefore, this secondary analysis of baseline data aims to explore factors across ecological levels (individual, interpersonal, environmental) associated with obesity among this population of women underrepresented in research.

## 2 Materials and methods

### 2.1 Study design

This study is a secondary cross-sectional analysis of baseline data from the HEALTH D&I study. A detailed description of HEALTH D&I methods can be accessed through the protocol paper (23). This study uses baseline data collected by research staff from a survey administered by telephone (during COVID) and on an iPad and in person (before COVID). Baseline data was collected from March 2019 to June 2022. Data collected in the survey ranged from demographic information, cardiometabolic health, health behaviors, food, and home environment, to social needs. The study was approved by the Washington University in St. Louis institutional review board.

### 2.2 Measures

#### 2.2.1 Dependent variable

The main outcome measure in this study was a binary measure of overweight (reference) compared to obese. Obesity was measured using self-reported height, and weight measured on an electric scale to calculate *body mass index (BMI)*. Weight was measured in person before COVID and over video call or phone with photos of the scale during COVID. BMI measures excess weight by calculating the weight in kilograms over height in meters squared (27). Based on BMI cut points, participants with BMI ranging from 25 kg/m^2 to 29.9 kg/m^2 were considered to be in the overweight BMI category and participants with BMI 30 kg/m^2 or more were considered to be in the obesity BMI category (27). Inclusion criteria for HEALTH D&I was BMI between 25 and 45.

#### 2.2.2 Independent variables

**Figure 1:**
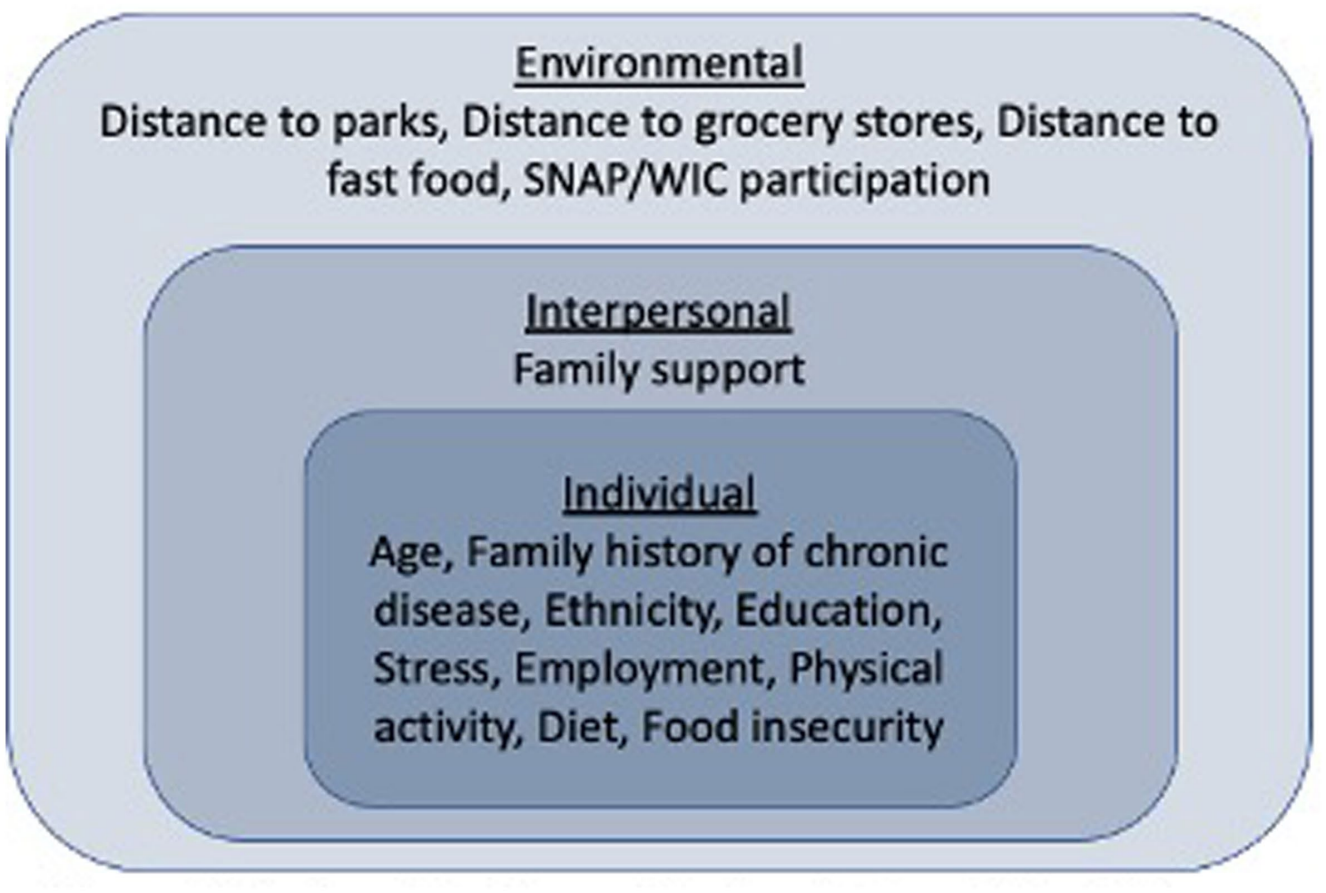
Socioecological model of predictors of obesity in women of reproductive age.

The independent variables in this study were chosen based on empirical research (e.g., systematic reviews) (3, 6, 17) around correlates of obesity and guided by the levels of the Socioecological model (individual, interpersonal, environmental). (Figure 1).

Individual level measures included *age* as a continuous variable (18–45)*, family history of chronic disease* (Yes, No/Not sure)*, ethnicity* (Hispanic/Latino, Not Hispanic or Latino)*, education* (High school graduate or less, Some college more), *stress* (High, Low)*, employment* (Employed, Not employed)*, physical activity* (High/Moderate, Low)*, dietary habits* as continuous variables (Ounces of whole grains a day, Teaspoons of added sugar a day, Cups of fruits and vegetables a day), and *food insecurity* (Food insecure, Food secure). Family history of chronic disease was based on whether participants reported a family history of diabetes, heart disease, or high blood pressure. Stress was measured using the perceived stress scale-4 (PSS-4) (28). The PSS-4 is the shortest version of the perceived stress scale, supporting use in surveys for minimizing respondent burden. This measure is widely used and though not as sensitive as the longer versions, has adequate reliability and strong criterion and construct validity (29–34). Cronbach’s alpha was 0.71. No cut points are indicated for high and low stress so the mean was used to dichotomize stress into a binary variable. Physical activity was measured using the International Physical Activity Questionnaire (IPAQ) (35). The IPAQ has been shown to have moderately high reliability and validity across diverse settings and populations (35–37). Additionally, the IPAQ has similar validity to other self-report measures (35). High physical activity represents an hour or more of activity a day that is at least of moderate intensity. Moderate physical activity is around half an hour of moderate physical activity most days. Low physical activity means the participant did not meet criteria for moderate or high physical activity (35). For analysis, physical activity was dichotomized into high/moderate and low. Dietary habits were measured using the National Health and Nutrition Examination Survey Dietary Screener Questionnaire (DSQ) which assess frequency of food (e.g., fruits, vegetables, whole grains, added sugar) and drink (e.g., sugar sweetened beverages) intake in the previous month (38). The DSQ has been widely tested and shows good validity when compared to other food recall measures (39–44). Food insecurity was measured using a two-item food insecurity measure developed by the United States Department of Agriculture Economic Research Service (45). The items have been tested for validity and reliability and are shown to accurately identify food insecurity (45–48). Survey items ask participants to indicate if in the last 12 months it was often true, sometimes true, or never true that “We worried whether food would run out before we got money to buy more” and “The food we bought just did not last, and we did not have money to get more.” (49) If participants answered “often” or “sometimes true” to both questions, they were considered food insecure.

The interpersonal level measure was *family support* as measured by the family support scale in the Home Environment Questionnaire (*α* = 0.63) (50). The family support measure was initially developed by Sallis et al. and has been used in the Home Environment Questionnaire with good validity and reliability for measuring social support specific to health behaviors (51–54). An example of family support questions include, in the last month how often did people living in your household do the following; encourage you to avoid unhealthy foods, discuss your eating habits with you, remind you to eat fruit and vegetables, and bring home foods you are trying to avoid. Participants responded with never/rarely, occasionally, often, or very often. The mean of all questions combined was used to create a continuous score, with higher scores indicating higher family support.

Environment level measures included *walking distance* (20 min or less, More than 20 min) *to the nearest fast food*, *grocery store*, *and park*, and participation in the Supplemental Nutrition Assistance Program (*SNAP*) and/or the Special Supplemental Nutrition Program for Women, Infants, and Children (*WIC*) (Yes, No/not sure).

### 2.3 Analysis

For the analysis, descriptive statistics were calculated for the outcome variable of BMI, and all independent variables for the total sample and for both BMI categories (overweight and obese). T-tests and Chi-squared tests were used to determine whether relationships between each independent variable and the outcome of BMI were significant. Variables that were significantly correlated with BMI, were included in a multivariate logistic regression model to determine the association of these variables with BMI. All quantitative analyses use Stata Version 17.

## 3 Results

In the analytic sample (*n* = 221), 37% of participants were overweight and 63% had obesity. (Table 1) The mean age was 31 (SD = 5.72) years old. Almost half of the sample (43%) identified as Hispanic or Latino and most (81%) reported a family history of chronic disease. Fifty-one percent of participants had a high school degree or less and 57% were not employed. Half of participants (50%) had low levels of physical activity, 43% had high stress, and 38% reported food insecurity. The mean predicted daily intake of added sugars was 18.4 (SD = 9.73) teaspoons, while the mean intake of whole grains was 0.58 (SD = 0.31) ounces, and mean intake of fruit and vegetables was 2.42 (SD = 0.65) cups. At the interpersonal level, mean score of family support was 1.72 (SD = 0.51). In terms of the environmental level variables, 52% of participants were within 20 min walking distance to the nearest fast-food outlet, while 58% had to walk more than 20 min to get to the nearest grocery store. The nearest park was more than a 20-min walk for 33% of participants. In total, 78% of participants participated in WIC and/or SNAP.

**Table 1:** Descriptive table of sample characteristics (*n* = 221).

	Sample	BMI overweight	BMI obese	*p*-value^a^
Sample		82 (37.10)	139 (62.90)	
Individual level^b^				
Age, m (SD)	30.70 (5.72)	29.82 (5.41)	31.22 (5.86)	0.08
Family history of chronic disease, n (%)				0.03
No/not sure	43 (19.46)	22 (26.83)	21 (15.11)	
Yes	178 (80.54)	60 (73.17)	118 (84.89)	
Ethnicity, n (%)				0.03
Not Hispanic/Latino	126 (57.01)	39 (47.56)	87 (62.59)	
Hispanic/Latino	95 (42.99)	43 (52.44)	52 (37.41)	
Education, n (%)				0.26
High school graduate or less	113 (51.13)	46 (56.10)	67 (48.20)	
Some college or more	108 (48.87)	36 (43.90)	72 (51.80)	
Stress, n (%)^c^				0.81
Low stress	127 (57.47)	48 (58.54)	79 (56.83)	
High stress	94 (42.53)	34 (41.46)	60 (43.17)	
Employment, n (%)				0.23
Employed	95 (42.99)	31 (37.80)	64 (46.04)	
Not employed	126 (57.01)	51 (62.20)	75 (53.96)	
Physical Activity, n (%)^d^				0.01
Low	110 (49.77)	32 (39.02)	78 (56.12)	
Moderate/High	111 (50.23)	50 (60.98)	61 (43.88)	
Predicted intake of whole grains (ounce equivalents) per day, m (SD)	0.58 (0.31)	0.58 (0.32)	0.57 (0.30)	0.89
Predicted intake of total added sugars (tsp equivalents) per day, m (SD)	18.4 (9.73)	17.73 (7.83)	18.78 (10.71)	0.44
Predicted intake of fruits and vegetables including legumes and French fries (cups) per day, m (SD)	2.42 (0.65)	2.53 (0.67)	2.36 (0.64)	0.07
Food insecurity, n (%)				0.03
Food secure	136 (61.54)	58 (70.73)	78 (56.12)	
Food insecure	85 (38.46)	24 (29.27)	61 (43.88)	
Interpersonal level^b^				
Family support, m (SD)^e^	1.72 (0.51)	1.68 (0.53)	1.74 (0.51)	0.37
Environmental level^b^				
Walking distance to nearest Fast food, n (%)				0.52
20 min or less	114 (51.58)	40 (48.78)	74 (53.24)	
More than 20 min	107 (48.42)	42 (51.22)	65 (46.76)	
Walking distance to nearest grocery store, n (%)				0.89
20 min or less	93 (42.08)	35 (42.68)	58 (41.73)	
More than 20 min	128 (57.92)	47 (57.32)	81 (58.27)	
Walking distance to nearest park, n (%)				0.67
20 min or less	147 (66.52)	56 (68.29)	91 (65.47)	
More than 20 min	74 (33.48)	26 (31.71)	48 (34.53)	
SNAP/WIC participation, n (%)				0.20
No/not sure	49 (22.17)	22 (26.83)	27 (19.42)	
Yes	172 (77.83)	60 (73.17)	112 (80.58)	

a *P-values based on t-test and chi-squared analyses.*

b Individual, interpersonal, and environmental levels modeled from socioecological framework.

c Stress measured with perceived stress scale and dichotomized at the mean.

d Physical activity measured with International Physical Activity Questionnaire (IPAQ).

e Family support measured with family support scale with scores ranging from 1 never/rarely to 4 very often.

Table 1 indicates results from bivariate analyses of each independent variable with the outcome of BMI category (likelihood of overweight or obesity). At the individual level, family history of chronic disease (x^2^(1) = 4.52, *p* = 0.03), ethnicity (x^2^(1) = 4.75, *p* = 0.03), physical activity (x^2^(1) = 6.03, *p* = 0.01), and food insecurity (x^2^(1) = 4.66, *p* = 0.03) were significantly associated with BMI category (27). None of the variables at the interpersonal or environmental levels were significantly associated with BMI.

The multivariate logistic regression model consisted of all four variables with significant associations in the bivariate analysis (family history of chronic disease, ethnicity, physical activity, and food insecurity); these were all four at the individual level (Table 2). This final model accounted for 7% (adjusted R(2) = 0.07) of variability in the odds of being in one BMI category or another. Family history of chronic disease and ethnicity were no longer statistically significantly associated with BMI category. Participants with high/moderate physical activity were 53% less likely to have obesity (OR = 0.47, 95% CI: 0.26,0.84, *p* = 0.01) than be overweight compared to participants who had low levels of physical activity. Participants who had food insecurity were 151% more likely to have obesity (OR = 2.51, 95% CI: 1.33,4.71, *p* = 0.00) than to be overweight compared to those who were food secure.

**Table 2:** Multivariate logistic regression model of correlates of BMI (Obesity vs. Overweight).

	OR	95% CI
Family history of chronic disease		
No/Not sure (reference)		
Yes	2.01	[0.95,4.25]
Hispanic/Latino		
Not Hispanic/Latino (reference)		
Hispanic/Latino	0.57	[0.31,1.03]
Physical Activity^a^		
Low physical activity (reference)		
Moderate/High physical activity	0.47^*^	[0.26,0.84]
Food insecurity		
Food secure (reference)		
Food insecure	2.51^**^	[1.33,4.71]
Observations	221	
Pseudo *R*^2^	0.07	

Exponentiated coefficients; 95% confidence intervals in brackets.^*^p < 0.05, ^**^p < 0.01, ^***^p < 0.001.

a Physical activity measured with International Physical Activity Questionnaire (IPAQ).

## 4 Discussion

In the present study, we applied the socioecological model to investigate individual, interpersonal, and environmental factors that are thought to be related to BMI status in a population of women of reproductive age underrepresented in research. We found that among this sample of women who are overweight or have obesity, individual level factors, but not interpersonal or environmental factors, were related to the risk of obesity (relative to overweight). Specifically, spending more time engaging in moderate or high intensity physical activity was associated with a lower risk of having obesity, while food insecurity was associated with a higher risk of having obesity. Our findings on physical activity and food insecurity are in line with previous research showing a relationship between physical activity and food insecurity with obesity (55, 56).

In our sample, 38% reported food insecurity and 78% received assistance from WIC and/or SNAP. This is important as we found that food insecurity was one of the strongest predictors of increased risk of obesity compared to overweight. This finding is similar to results from previous studies (56), and supports research which has explored mechanisms through which food insecurity can increase risk of obesity (57–59). Our results highlight the continued importance of assessing and addressing SDOH as a method of decreasing obesity risk, especially among those who identify as Hispanic/Latino and who are in low-socioeconomic positions. In our sample, 43% identified as Hispanic/Latino and 51% had an education level of high school graduate or less. It should be noted that the question in our study assessed food insecurity at the individual level (e.g., ability to afford food). However, our finding may reflect SDOH or environmental level determinants such as economic stability and neighborhood food and physical environment (14), beyond what was measured in the current study with walking distance to the nearest park, fast food, and grocery store. For example, the ability to afford enough healthy food can be driven by SDOH such as economic stability (e.g., employment, education, debt) as well as access, acceptability, and availability of healthy affordable food (e.g., neighborhood food environment) (60).

Time spent engaging in high/moderate physical activity was associated with a lower risk of having obesity. The association between physical activity with a reduced risk of obesity is evident in previous research (55). Our findings add support that in an ethnically and socioeconomically underrepresented population of women of reproductive age, engaging in physical activity, is related to reduced weight. This finding is important for obesity prevention efforts among women of reproductive age. While 50% of participants in our study reported moderate/high physical activity, previous research indicates physical activity may decrease with parenthood (61).

Our results that the interpersonal and environmental level factors were not related to the risk of obesity (relative to overweight) are different from previous research which finds interpersonal factors (e.g., social support) and environmental level factors (e.g., physical activity and healthy food opportunities in the neighborhood) are associated with obesity (6, 14, 60, 62). These different results may be due to how interpersonal and environmental level factors were measured. Specifically, the physical activity and food environment was measured through a single metric of distance to the nearest park, grocery store, and fast-food outlet. Other interpersonal and environmental factors such as walkability, safety, social norms around eating and activity, neighborhood aesthetics, and public transit were not included. Previous research underscores the complexity of measuring environmental level determinants of obesity and health behaviors (63–65). In particular, it is challenging to tease apart the cumulative and interacting elements of the neighborhood environment and ways these elements influence behavior.

## 5 Limitations

Our study is not without limitations. We used BMI which is an indirect measure of obesity and therefore may not accurately determine overweight or obesity at the individual level (66). BMI is also modeled on white adults and may not necessarily translate to other races and ethnicities, introducing bias into the measure (27). This is especially salient for this study in which 43% of the participants identify as Hispanic and Latino. This was a cross-sectional analysis so it does not allow for causal determinations, but our findings do provide support for future longitudinal and/or experimental studies to investigate how modifiable factors such as food insecurity and physical activity could reduce obesity risk. We had limited measures at the environmental level (e.g., walking distance to nearest park, grocery store, and fast-food outlet), and broader factors like racism and discrimination, which could influence the results, were also not measured. We used the perceived stress scale to measure stress among participants in the study. While this scale has been validated for use in diverse populations, it may not adequately assess the stress experienced by populations who have experienced marginalization (67). Our small sample size and secondary analysis may have reduced our ability to detect significant associations. Lastly, due to an ICC of less than 0.01 for the outcome of BMI, we were unable to run a multilevel model.

## 6 Conclusion

In conclusion, our findings support that food insecurity and physical activity may be important factors associated with obesity relative to overweight in women of reproductive age. Interventions and policies for addressing obesity in this population may be effective by targeting health behaviors like physical activity and drivers of food insecurity. Our study contributes knowledge about obesity risk across ecological levels for women who identify as Hispanic/Latino and those of low-socioeconomic status of reproductive age, a population that is less represented in research.

## Data Availability

The datasets used/analyzed during the current study are available from the corresponding author on reasonable request.
